# Ecosystem-scale genomic and metatranscriptomic evidence for a coupled, active short-term hydrocarbon cycle in the marine photic ocean

**DOI:** 10.1128/aem.01045-26

**Published:** 2026-07-21

**Authors:** Kathryn L. Howe, Olivia U. Mason

**Affiliations:** 1 Department of Earth, Ocean and Atmospheric Science, Florida State University 7823, Tallahassee, Florida, USA; University of Nebraska-Lincoln, Lincoln, Nebraska, USA

**Keywords:** archaea, bacteria, microbial, genomics, metatranscriptomics, hydrocarbon cycle, microbes

## Abstract

Natural hydrocarbon seeps and human activities are the primary pathways by which oil enters the sea. Marine Cyanobacteria, among the most abundant photosynthetic organisms in the world, continuously produce alkanes, an additional substantial hydrocarbon source to the sea. These alkanes form the basis of the short-term hydrocarbon cycle (STHC). Alkane production proceeds via fatty acyl-ACP reductase (*faar*) and aldehyde deformylating oxygenase (*ado*), and degradation occurs via alkane monooxygenases (*alkB/cyp153*). Although alkane biosynthesis and degradation pathways have been characterized, ecosystem-scale evidence for their concurrent expression *in situ* is lacking. Here, we integrated genomic annotation and analysis of >9,000 marine bacterial and archaeal genomes representing globally distributed microbial lineages, including cultured isolates, and predominantly uncultured taxa, with metatranscriptomic read mapping. Expression of *faar* and *ado* was dominated by *Prochlorococcus* and, to a lesser extent, *Synechococcus*. Bacterial alkane consumption via *alkB* was dominated by Pseudomonadota, SAR324, and Bacteroidota. Archaeal alkane consumption utilizing this same degradation pathway was observed in the transcript data by Thermoplasmatota, classified as Marine Group II and III, the most abundant planktonic archaeal groups. Active production of alkanes in the photic zone would be an important component of the marine hydrocarbon cycle, and more broadly, of carbon cycling. Furthermore, consumption of hydrocarbons, regardless of source, is a fundamentally important ecosystem cleanup service provided by microbes in the ocean. The simultaneous transcription of alkane biosynthesis and degradation genes provides ecosystem-scale evidence for a coupled and active microbial STHC that is independent of external petroleum inputs.

**IMPORTANCE:**

Hydrocarbons in the long-term hydrocarbon cycle (LTHC) are produced over long time scales, whereas cyanobacterial *n*-alkane production occurs on short time scales in the short-term hydrocarbon cycle (STHC). Continuous *in situ* cyanobacterial production contributes >100 million tons of hydrocarbons annually to the ocean. Despite differences in source and timescale, these cycles are linked by microbes that degrade *n*-alkanes using the same pathways. Here, we demonstrate that alkane production and degradation genes are concurrently expressed in the sunlit ocean, providing evidence for an active STHC based on annotation of >9,000 genomes representing globally distributed microbial lineages, including predominantly uncultured taxa, and metatranscriptomic read mapping. Importantly, an active STHC may support a population of alkane-degrading bacteria and archaea, enabling a rapid response to oil input from a geological reservoir as part of the LTHC, through natural or anthropogenic activities, with implications for bioremediation and marine ecosystem health.

## INTRODUCTION

Microbes play a pivotal role in marine hydrocarbon degradation, a critical ecosystem service for the remediation of oil spills and natural seepage that is prevalent throughout the Gulf of Mexico (GOM; historical name used here for consistency) and the global ocean. The long-term hydrocarbon cycle (LTHC), the process in which oil that is millions of years old is released into the marine environment, either naturally or by drilling and transportation operations, and consumed by microbes, is best exemplified through the bioremediation by planktonic microbes during the 2010 GOM Deepwater Horizon (DWH) oil spill.

In contrast, the rapid cycle of cyanobacterial production of hydrocarbons and consumption by microbes occurs over days and is termed the short-term hydrocarbon cycle (STHC). For over 50 years, Cyanobacteria have been known to produce the raw materials for hydrocarbon fuels (1–3) in the form of medium- and long-chain alkanes (4). Roughly 308–771 million tons of these alkanes, specifically pentadecane (C_15_) and heptadecane (C_17_), are produced annually by cyanobacteria, which is ~1% of the bioavailable dissolved organic carbon in the ocean (5, 6) and ~100- to 500-fold more than total oil input into the ocean from all sources (6, 7). The functional role of these hydrocarbons in Cyanobacteria was revealed by *in vivo* measurements, which showed distortions in cell morphology in knock-out mutants compared to wild-type *Synechococcus*; this suggests that these hydrocarbons are required for optimum cell division, size, and growth (8). Additionally, they may play a role in response to salinity (9), cold stress (10), and redox balance (10).

To date, all cyanobacterial genomes encode hydrocarbon production (11), mainly C_15_–C_17_ alka/enes (5). This is done primarily via the FAAR/ADO pathway (4), which proceeds via metabolism of fatty acid intermediates with a fatty acyl-ACP reductase (FAAR) and an aldehyde-deformylating oxygenase (ADO) (12). Lea-Smith et al. (5) also showed that alkanes produced by Cyanobacteria were able to support a population of *Alcanivorax borkumensis*, a gammaproteobacterium considered a paradigm of *n*-alkane degradation in the marine environment (13, 14). Love et al. (6) assembled metagenomes from shipboard incubations with pentadecane and reported ~20 bacterial genomes encoding the potential for *n*-alkane degradation, with *Alcanivorax* dominating incubations.

Hydrocarbon degradation by heterotrophic microorganisms was demonstrated more than 100 years ago (15), and over 75 genera of hydrocarbon-degrading bacteria have since been recognized (16). In fact, hydrocarbon degradation by marine microorganisms is cited as the reason the ocean is not covered in a layer of oil accumulated from millions of years of natural seepage (16). Recently, Howe et al. (17) presented novel metagenome-assembled genome (MAG) and metatranscriptomic read mapping data showing that uncultured Gammaproteobacteria are primarily responsible for degrading aliphatic and aromatic hydrocarbons in the global ocean. These alkane degradation pathways primarily proceed via alkane 1-monooxygenase (AlkB) and/or cytochrome P450 alkane hydroxylase (CYP153).

Although hydrocarbon production by marine Cyanobacteria has been shown both in the laboratory by Lea-Smith et al. (5, 8), and in shipboard incubations by Love et al. (6), the identity and function of *in situ* producers were not reported, nor was transcriptional activity provided. Furthermore, Love et al. (6) did not present transcript data on microbial consumption of *n*-alkanes. Whether these processes are concurrently active at the ecosystem scale remains unresolved. To date, *in situ* alkane production and consumption pathways have not been attributed to specific marine microbes within the same ecological context of the sunlit ocean. Moreover, the participation of uncultured taxa in an active STHC has not been assessed. To address this knowledge gap, we integrated annotation of >9,000 marine bacterial and archaeal genomes, including cultured isolates and predominantly uncultured taxa from the global ocean, with metatranscriptomic read mapping from an active GOM seep site. This approach allowed us to evaluate both the genomic potential and *in situ* expression of alkane biosynthesis and degradation pathways across diverse microbial groups. By examining concurrent transcription of production and consumption genes, we tested the hypothesis that the marine photic zone sustains a coupled STHC in the sunlit ocean and explored linkages with the LTHC.

## RESULTS

### Hydrocarbon producers, read recruitment, and genome similarity

**Fig 1:**
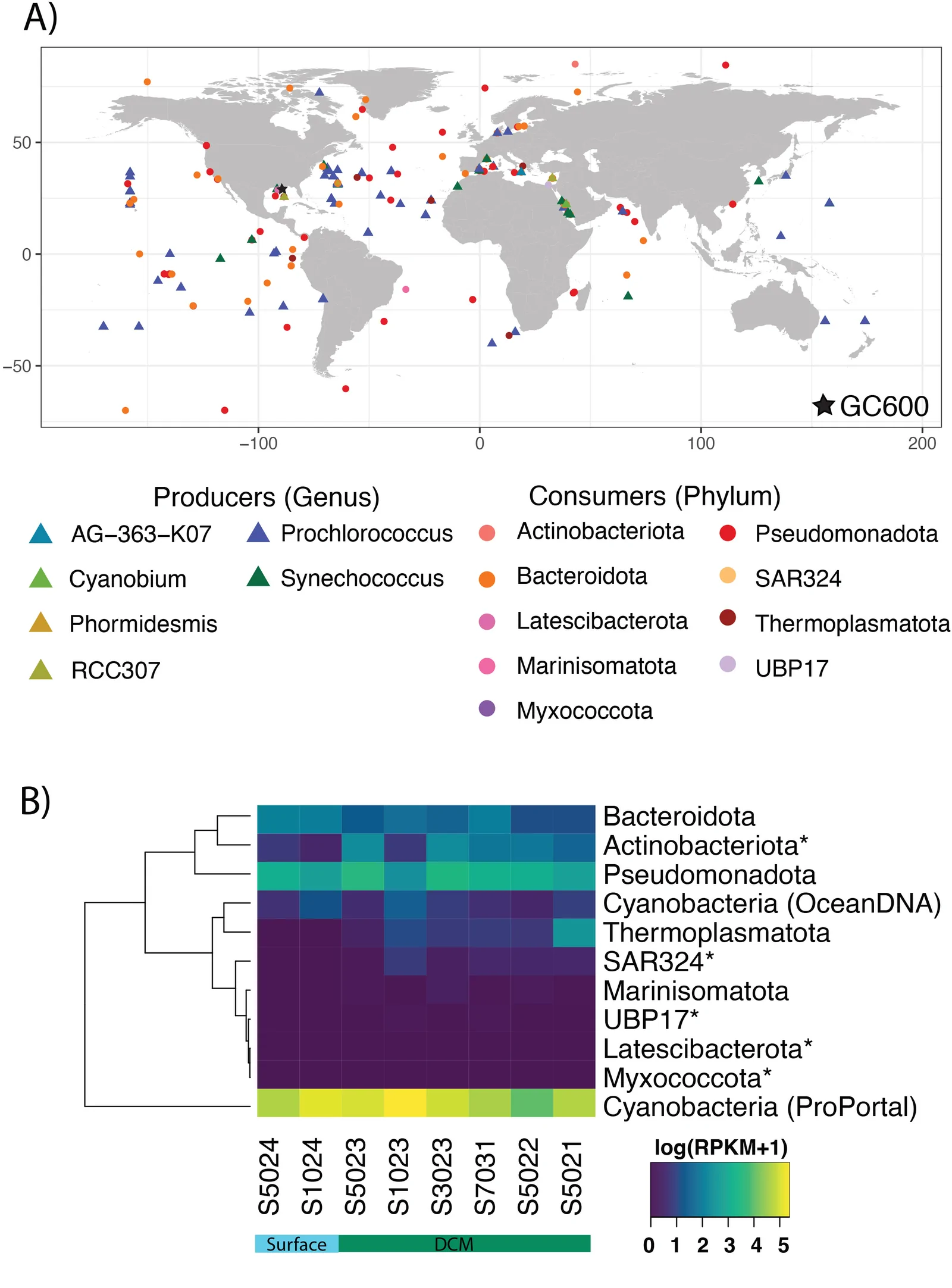
(**A**) Map showing sample locations for the 506/9,379 microbes in this study that expressed at least one alkane production or consumption gene. Color corresponds to clade, and shape indicates if the microbe is an alkane producer (triangle) or consumer (circle). The GC600 sample site is marked with a star. The map was created using Ocean Data View (ODV) software. (**B**) Genome-level read recruitment for all microbes that express at least one alkane production/consumption gene summed by phylum, with Cyanobacteria separated by database.

Analyses of 9,379 ProPortal and OceanDNA genomes (Tables S1 and S2) revealed *faar*/*ado* (E.C. 1.2.1.80 and 4.1.99.5, respectively) genes in 570/913 (62%) ProPortal genomes and 108/8,466 (1%) OceanDNA MAGs. Of these 678 genomes/MAGs encoding alkane-production genes, 341 recruited metatranscriptomic reads to *faar* and/or *ado*, including 320 ProPortal genomes and 21 OceanDNA MAGs. These genomes were classified as Cyanobacteria, Actinobacteriota, Myxococcota, Bacteroidota, Pseudomonadota, and Udinarchaeota. Only those genomes with production genes that were expressed were analyzed further. Read mapping revealed 604 *faar*/*ado* genes were expressed by 341/678 (50%) microbes as revealed by mapping GOM metatranscriptome reads (Fig. 1; Tables S1 and S3). These genomes were classified as belonging to *Cyanobium*, *Prochlorococcus*, *Synechococcus*, RCC307, AG363K07, or *Phormidesmis* (Fig. 1; Tables S1 and S3).

Specifically, of 913 *Prochlorococcus* and *Synechococcus* genomes in ProPortal, 1,113 *faar* and *ado* sequences were identified in 570/913 microbes, which were sampled from a variety of locations in the global ocean, including epipelagic, pelagic, oceanic, marginal sea, and intertidal zones, and contained 92 cultured representatives (Fig. 1; Table S1). Read mapping revealed 575/1,113 (52%) of these genes were expressed and encoded in 320 microbes, which were included in all subsequent analyses (Tables S1 and S3). Of these 320 ProPortal microbes, 254/320 (79%) encoded both *faar* and *ado*, 28 encoded only *faar*, and 38 encoded only *ado*. These same analyses revealed additional genomes encoding *faar*/*ado* genes, some of which were expressed, from the 8,466 prokaryotic species-level clusters in the OceanDNA MAG catalog (18). Specifically, 214 alkane production genes in 108/8,466 (1.3%) MAGs in six different phyla (Cyanobacteria, Actinobacteriota, Myxococcota, Bacteroidota, Pseudomonadota, and Udinarchaeota) were identified. Ultimately, 29/214 (14%) genes from 21/108 (19%) MAGs from one phylum (Cyanobacteria) were expressed. Therefore, these 21 cyanobacterial MAGs were included in all other analyses (Fig. 1; Table S4).

**Fig 2:**
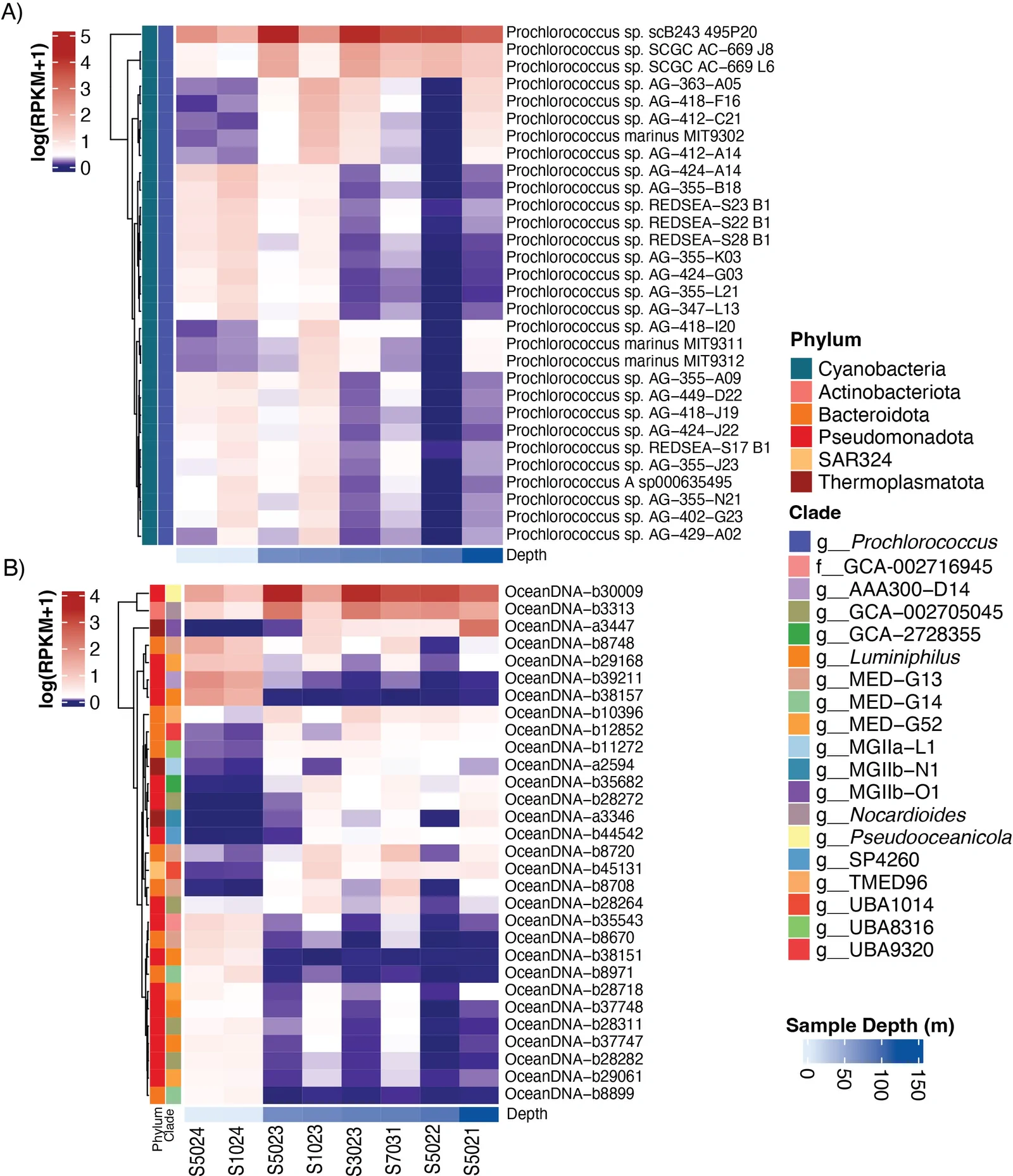
Genome-level read recruitment for the top 30 alkane producers (**A**), and top 30 alkane consumers (**B**). Samples are arranged by increasing depth from left to right, shown by the depth color bar. The left vertical color bar corresponds to the phylum, while the right vertical color bar indicates clade.

Genomes were grouped by phylum, revealing that cyanobacterial read recruitment was the highest of all phyla, with summed phylum reads per kilobase per million (RPKM) ranging from 53 to 217 (Fig. 1B), inclusive of hydrocarbon consumers. Specifically, based on read recruitment for individual genomes, *Prochlorococcus* sp. scB243 495P20, assembled from a Bermuda Atlantic Time Series (BATS) site, with a whole-genome RPKM of 70 at one depth (Fig. 2A), and two *Prochlorococcus* sp. SCGC AC-669 (J8 and L6), assembled from off the coast of Chile (Fig. 2A), had the highest read recruitment in the entire data set.

Genome comparison revealed that the average nucleotide identity (ANI) among the alkane producers, which were all Cyanobacteria, was >80% (Fig. S1). *Prochlorococcus* had a high within-clade similarity, with many of the *Prochlorococcus* MIT strain genomes having 100% similarity to each other. The within-clade ANI maximum for *Synechococcus* was 99.3% between *Synechococcus* E sp002724845 (OceanDNA-b16691) and *Synechococcus* sp. REDSEA-S02 B4 (Fig. S1). The single *Cyanobium* MAG (OceanDNA-b16273) typically had higher similarity with *Prochlorococcus* than with *Synechococcus*, while the single AG-363-K07 MAG (OceanDNA-b16239) had the opposite relationship. The single *Phormidesmis* MAG (OceanDNA-b16232) had less than 75% similarity to any other hydrocarbon producer analyzed herein (Fig. S1). Last, the two MAGs in the RCC307 clade were 82% similar to each other, with one having a 99% similarity with two *Synechococcus* genomes (Fig. S1).

### Expression of microbial hydrocarbon production genes

**Fig 3:**
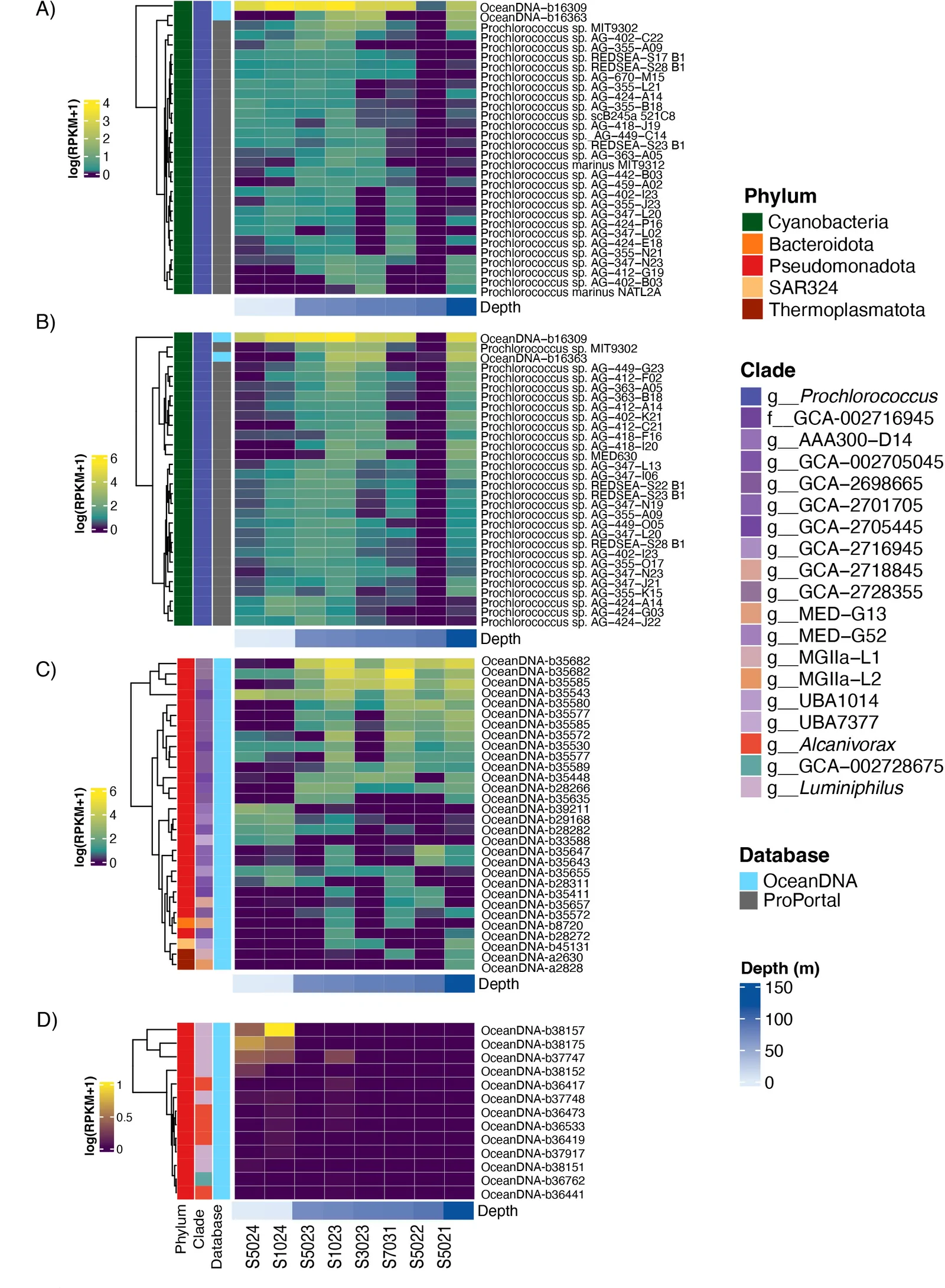
Gene-level read recruitment for the top 30 microbes that expressed each primary alkane production or consumption gene: *faar* (**A**), *ado* (**B**), *alkB* (**C**), and *cyp153* (**D**), except for *cyp153*, which was expressed by only 13 microbes. Samples are arranged by increasing depth from left to right, shown by the depth color bar. The left vertical color bar corresponds to the phylum, the middle vertical color bar indicates clade, and the right vertical color bar indicates database.

Transcript levels of *ado* and *faar* in the GOM metatranscriptomes were highest for OceanDNA MAG *Prochlorococcus A* sp000635495 (OceanDNA-b16309) assembled from the Red Sea, with an RPKM maximum of 266 and 41, respectively. When summed across all depths, RPKM transcript values were 964 for *ado* and 177 for *faar* for this organism (Fig. 3A and B). Seven other cyanobacterial MAGs from the OceanDNA MAG catalog encoded both *faar* and *ado* and recruited reads to one or both genes. Of these, *Prochlorococcus B marinus B* (OceanDNA-b16363) assembled from a GEOTRACES sample in the Mediterranean had the next highest *ado* expression, with a summed RPKM of 105 across all depths and RPKM >30 in three deep chlorophyll maximum (DCM) samples, which were defined in Rakowski et al. (19). In this organism, *faar* expression was up to an RPKM maximum of 7.4 at one DCM depth (Fig. 3A and B). *Prochlorococcus* sp. MIT9302 had the highest *ado* and *faar* gene expression levels for any microbe in ProPortal, with summed expression across all depths of 68 and 13 RPKM, respectively (Fig. 3A and B).

*Synechococcus* genome and gene recruitment were typically much less than that of *Prochlorococcus* genomes and genes (Fig. 3A and B). *Synechococcus ado* expression was highest for *Synechococcus* sp. CC9605 with an RPKM maximum of 3, which was the second highest recruitment to any alkane production gene at 82 mbsl by any ProPortal genome. *faar* expression was highest for *Synechococcus* sp. MED850, with an RPKM maximum of 0.94 in a surface sample.

### Hydrocarbon consumers, read recruitment, and genome similarity

Using the same approach discussed above, alkane consumers were evaluated, revealing 2,885 *alkB*/*cyp153* (E.C. 1.14.15.3) genes from 2,251/8,466 (27%) MAGs in the OceanDNA MAG catalog spanning 26 phyla, with gene expression observed in nine of these phyla (Fig. 3C and D; Tables S2 and S5). Altogether, 173 alkane hydroxylase (*alkB* and *cyp153*) genes were expressed in 165 (7%) MAGs, which were classified as Actinobacteriota, Bacteroidota, Latescibacterota, Marinisomatota, Myxococcota, Pseudomonadota, SAR324, UBP17 in the Bacteria, and Thermoplasmatota in the Archaea (Fig. 3C and D; Tables S2 and S5). Additionally, the GOM metatranscriptomic reads were mapped to the genes annotated as *alkB*/*cyp153* presented in Love et al. (6), but none recruited any reads and therefore were not analyzed further.

Genome read recruitment revealed that Pseudomonadota recruited the greatest number of reads of any consumer (RPKM range 10–33), followed by Actinobacteriota, Bacteroidota, and Thermoplasmatota (Fig. 2B, 3C and D). The highest read recruitment for individual alkane consumers was from an alphaproteobacterial MAG in the order Rhodobacterales (OceanDNA-b30009) with an RPKM range 0.6–9.1, followed by an Actinobacteriota MAG, a member of the archaeal Thermoplasmatota (OceanDNA-a3447) assembled from the Hawaiian Ocean Time-series data, and a Pseudomonadales (OceanDNA-b39211) assembled from Hawaii sea surface (Fig. 2B). All remaining alkane consumer genomes had RPKM values of <5 (Fig. 2B).

Given that alkane consumers were identified across several different phyla, ANI was low (Fig. S2). For example, Latescibacteriota, Myxococcota, SAR324, UBP17, Marinisomatota, and Actinobacteriota MAGs were <77% similar to any other MAG. In contrast, within-clade similarity was much higher, as exemplified by Pseudomonadota, and particularly the Gammaproteobacteria, which were most similar to each other. In this clade, the highest similarity (97%) was observed between two *Alcanivorax* genomes, *Alcanivorax* sp. 002354605 (OceanDNA-b36419) and *Alcanivorax* sp. 002354605 (OceanDNA-b36533; Fig. S2). Bacteroidota also had high within-clade similarity of up to 95% (Fig. S2). Finally, the archaeal within-clade similarity among Thermoplasmatota MAGs was up to an ANI of 93% (Fig. S2).

### Expression of microbial hydrocarbon consumption genes

Among alkane consumers, Pseudomonadota showed the highest read recruitment to alkane-consumption genes, consistent with relatively high transcriptional activity for these pathways in this data set (Fig. 2B, 3C and D). A total of 527 Gammaproteobacteria MAGs encoded 765 alkane hydroxylase genes, with 74 genes from 66 MAGs in 19 orders recruiting reads. These included GCA-002705445, Pseudomonadales, Burkholderiales, UBA4575, Ga0077536, UBA10353, Woeseiales, SAR86, Immundisolibacterales, Nevskiales, and Nitrosococcales. *AlkB* expression was highest in GCA-002705445 microbes, from which 37 *alkB* genes were encoded, with four genes having RPKM values >10 (Fig. 3C). In this clade, one MAG expressed two copies of its *alkB* gene, both with a summed RPKM of all depths of over 200, with the highest expression in DCM samples (RPKM was 4–177; Fig. 3C). Other members of this clade had similar, yet slightly lower *alkB* expression (Fig. 3C), with Pseudomonadales, the Porticoccaceae, Oleiphilaceae, and Spongiibacteraceae families recruiting reads to their *alkB* genes but at lower levels than GCA-002705445 microbes. Interestingly, none of the *alkB* encoded by canonical gammaproteobacterial *n*-alkane degraders, such as *Bermanella* (three MAGs), *Cycloclasticus* (eight MAGs), *Oleispira* (eight MAGs), and *Colwellia* (one MAG), were expressed, except for one *Alcanivorax* (14, 17, 20–27). Pseudomonadota, specifically the Pseudomonadales (Halieaceae, Alcanivoracaceae, and HTCC2089), was the only clade to express *cyp153* genes, but expression levels were low compared to *alkB* expression (Fig. 3C and D).

Altogether, 365 archaeal *alkB* genes were encoded in 361 Thermoplasmatota MAGs, all from Marine Group II (MGII) or Marine Group III (MGIII) in the class Poseidoniia. Of the 365 *alkB* genes, 14 recruited metatranscriptomic reads (Fig. 3C; Table S5). *Poseidoniaceae* MGIIa-L2 sp00249919*5* (OceanDNA-a2828) had the highest *alkB* expression by any archaea (RPKM maximum value of 1.5), followed by *Poseidonia* sp002714305 (OceanDNA-a3447; RPKM maximum of 1.3). MGIIa-L1 sp8160u (OceanDNA-a2630) recruited metatranscriptomic reads to its *alkB* genes at the most depths (three depths with RPKM range 0.05–1.23) with the remaining archaeal *alkB* genes having minimal expression. Only one archaeal MAG (OceanDNA-a3413) expressed *alkB* in a surface sample (RPKM was 0.02), whereas the rest of the activity was in the DCM.

### Secondary alkane consumption genes

Secondary alkane consumption genes, including generic cytochrome P450 (not annotated as an alkane hydroxylase; *cyp450*, E.C. 1.14.14.1) and ferredoxin (E.C. 1.18.1.2), were expressed by Actinobacteriota and Bacteroidota, while only Bacteroidota expressed rubredoxin (E.C. 1.18.1.1; Fig. S3). Flavin-binding monooxygenase (*fmo*, E.C. 1.14.13.8) genes were expressed by Bacteroidota, Myxococcota, Planctomycetota, Pseudomonadota, and Thermoplasmatota. Although ferredoxin reductase genes were encoded, none recruited any reads.

Other genes involved in consumption, such as *fmo* (E.C. 1.14.13.8), were observed in the Pelagibacterales with six MAGs encoding *fmo* and three expressing it (Fig. S3). In addition to the Pelagibacterales MAGs, 10 other Alphaproteobacteria expressed *fmo*, with an RPKM maximum of 8 by *Rhizobales* AG-430-B22 sp003212795 (OceanDNA-b27101; Fig. S3). Other microbes classified as Flavobacteriales SAR86, Ga0077536, and one Myxococcota encoded and expressed *fmo*, some of which also expressed ferredoxin (RPKM max of 3.6), and *cyp450* (RPKM maximum of 0.04; Fig. S3). Only one Thermoplasmatota MAG expressed *fmo* (RPKM < 0.04), but no other secondary alkane consumption genes. Actinobacteriota and Bacteroidota MAGs expressed *cyp450* with an RPKM maximum of 1.76 (Fig. S3). One Planctomycetota classified as Pirellulales (OceanDNA-b22322) expressed *fmo* with an RPKM <0.02, yet did not express any other alkane consumption genes. Only one Bacteroidota MAG classified as Flavobacteriales (OceanDNA-b9761), which expressed rubredoxin, expressed another alkane consumption gene, in this case ferredoxin, yet both were minimally expressed (RPKM < 0.05; Fig. S3).

## DISCUSSION

To evaluate transcriptional activity in the STHC and explore linkages with the LTHC, we selected the Green Canyon 600 (GC600) lease block in the GOM, an area of natural hydrocarbon seeps (28) with persistent surface oil slicks (28, 29). However, slicks resulting from GC600 seepage are characterized as highly weathered, with *n*-alkanes < C_15_ being largely absent, and C_16_–C_34_ components comprising 3%–8% of the *n*-alkane pool (30). Furthermore, at GC600, surface oil slicks are ephemeral, with an average residence time of approximately 6.4 h. The depletion of *n*-alkanes < C_15_ coupled with the ephemeral nature of oil slicks at this site may explain why DWH microbes described in Howe et al. (17) were inactive at GC600, suggesting that while the LTHC and STHC are linked through metabolic pathways for consumption, sustaining a population of hydrocarbon-degrading microbes is likely supported by alkane production in the STHC. To this end, it is estimated that alkanes produced as part of the STHC would represent an additional hydrocarbon source in the global sunlit ocean that could equate to 308–771 million tons of hydrocarbons input annually (5), an amount which exceeds that of hydrocarbons produced by Saudi Arabia (31). This additional substantial input of hydrocarbons outside of the LTHC could sustain populations of alkane-degrading microbes, such as those presented herein, and would facilitate rapid microbial consumption of alkanes from the LTHC.

By integrating the complete annotation of over 9,000 global genomes with metatranscriptomes from GC600 in the Gulf of Mexico, we show both sides of microbial hydrocarbon production and consumption using genome-resolved and transcriptional evidence in the same system. Our results show the first *in situ* expression of genes in the STHC and reveal globally distributed microbes with genomic potential for this process and demonstrate transcriptional activity for representative lineages in the GC600 photic-zone metatranscriptomes, suggesting active expression rather than latent genomic potential. For example, *Prochlorococcus* recruited the most reads among alkane producers, with the highest overall *faar* and *ado* gene expression levels. In particular, two uncultured *Prochlorococcus* assembled from the Red Sea and from a BATS sample recruited the greatest number of reads to their *faar* and *ado* genes. In laboratory experiments with cultured *Prochlorococcus*, Lea-Smith et al. (5) measured hydrocarbon production by *Prochlorococcus* MIT9312 (high-light adapted strain and one of the most numerically abundant *Prochlorococcus* ecotypes) and MIT9313 (low-light adapted strain), both of which were determined to actively express alkane production genes *in situ* in our samples. Lea-Smith et al. (5) also showed that *Synechococcus* produces *n*-alkanes, consistent with our findings that members of this group encode and express *faar* and *ado* genes *in situ*. Specifically, they showed that *Synechococcus sp*. WH8102, WH7803, and WH7805 produce *n*-alkanes. All three were found to encode *faar* and *ado* in our samples, yet only *Synechococcus sp*. WH8102 expressed these genes *in situ*. Thus, our findings provide the missing information needed to link laboratory observations to *in situ* gene expression for *n*-alkane production pathways as part of the STHC. Furthermore, inclusion of uncultured microbes from the global ocean that were actively transcribing genes involved in alkane production broadens our understanding of how widespread alkane production is in the marine environment.

In terms of *n*-alkane consumption, Pseudomonadota dominated alkane-consumer read recruitment, with the highest *alkB* transcript recruitment. In particular, members of the gammaproteobacterial order GCA-002705445 had the highest *alkB* expression levels. This order contains methylotrophs that have been shown to increase in abundance in response to phytoplankton blooms (32). Additionally, Porticoccaceae MAGs assembled from a low-oxygen Hawaiian Ocean Time-series sample, the North Sea, and the Mediterranean Sea expressed *alkB*. Members of this group have also been shown to be associated with phytoplankton and are known to degrade PAHs (33–35). Other Pseudomonadota, such as Rhodobacteraceae in the Alphaproteobacteria, encoded and expressed *alkB*. Rhodobacteraceae are known to degrade aromatic hydrocarbon compounds (36), to colonize plastics (37, 38), to be present and active in hydrocarbon-contaminated environments (39, 40), and were the predominant bacteria affecting *n*-alkane concentrations in DWH microcosms presented in Hu et al. (41). Overall, our findings provide greater resolution on why these microbes that can degrade alkanes, such as those produced by phytoplankton as part of the STHC, may respond to phytoplankton blooms. Furthermore, the ability to degrade hydrocarbons as observed through genome analysis and read mapping extends our understanding of why members of these clades are present in hydrocarbon-contaminated environments and what type of hydrocarbons they have the genetic machinery to degrade.

Outside the Pseudomonadota, Bacteroidota, specifically the Flavobacteriales, Actinobacteriota, Latescibacterota, SAR324, Myxococcota, and UBP17 members encoded and expressed *alkB*, with Flavobacteriales being the only group to express all secondary alkane consumption genes and the only group to express rubredoxin. Members of these clades are known to degrade aromatic and aliphatic hydrocarbons, are often associated with the plastisphere, and some are also known to increase in abundance in response to a phytoplankton bloom (6, 17, 34, 36, 42–48). Thus, our understanding of these microbes is largely that they respond to oil input as part of the LTHC, but our results suggested that, like the Pseudomonadota, they may also play an important role in the consumption side of the STHC.

Archaeal alkane consumption by Thermoplasmatota (class Poseidoniia) using *alkB* was observed in the transcript data. These MAGs were from the Atlantic and Pacific Oceans, as well as the GOM and Mediterranean Sea. Poseidoniia includes MGII and MGIII, which are the most abundant planktonic archaeal groups in the surface ocean water column (49) and are frequently observed during or after phytoplankton blooms due to possible syntrophic interactions between these groups (50). The limited number of sequenced genomes and the lack of cultured representatives of Thermoplasmatota, specifically MGII and MGIII subgroups, leave a knowledge gap about their metabolic capabilities; however, they have previously been shown to be capable of alkane degradation with *alkB* (34, 46, 49), and some of these sequences cluster with gammaproteobacterial *alkB* (34). Additionally, analysis of TARA Oceans data by Love et al. (6) revealed a clade of *alkB*-like monooxygenases belonging to MGII/MGIII that was persistent in all surface and DCM samples of the Northern Atlantic Ocean, suggesting that members of these MGII and MGIII clades may play a role in the STHC. Our study provides new, important information on active transcription of alkane consumption genes in Thermoplasmatota, specifically in MGII and MGIII, that appear to be involved in both the LTHC and STHC.

Collectively, our analysis of >9,000 marine genomes, integrated with metatranscriptomic read mapping, provided lineage-resolved evidence for concurrent expression of alkane production and degradation pathways within the STHC. Given the substantial quantities of hydrocarbons produced by marine Cyanobacteria, continuous microbial consumption is required to prevent their accumulation in surface waters. Furthermore, these hydrocarbons could support a population of microbes that are poised to degrade hydrocarbons from the LTHC as they transit through the marine water column. Thus, microbes described here could play an important role in marine health as they are able to consume hydrocarbons produced by Cyanobacteria, natural seepage, and anthropogenic input. Finally, linking functional potential and *in situ* transcription to the specific bacterial and archaeal players, including uncultured taxa, reveals candidate organisms and pathways that can now be targeted for physiological characterization and cultivation efforts.

## MATERIALS AND METHODS

### Sample collection

Eight depths at GC600, an active seep with visible oil at the surface, in the GOM were sampled during an expedition in June 2013 on the *R/V Pelican* (19). Collection depths spanned the near-surface (<2 mbsl) to 120 mbsl, targeting the euphotic zone and DCM. The microbial communities at this site were described in Rakowski et al. (19). These samples from eight depths at GC600 were selected for metatranscriptomic sequencing.

### Microbial sample collection and RNA extractions

From GC600, 10 L of seawater was collected from eight depths and filtered with a peristaltic pump. A 2.7 μm Whatman GF/D pre-filter was used, and samples were concentrated on 0.22 μm Sterivex filters (EMD Millipore). Sterivex filters were sparged and filled with RNAlater. RNA was extracted directly from the filter by placing half of the Sterivex filter in a Lysing matrix E tube containing glass, zirconia, and silica beads (MP Biomedicals, Santa Ana, CA) using the protocol described in Gillies et al. (51), which combines phenol:chloroform:isoamyl alcohol (25:24:1) and bead beating. RNA was stored at −80°C until purified using QIAGEN (Valencia, CA) AllPrep DNA/RNA Kit.

### Metatranscriptomic sequencing

For metatranscriptomic sequencing, only RNA with an RNA integrity number (16S/23S rRNA ratio determined with the Agilent TapeStation) ≥9.5 (on a scale of 1–10, with 1 being degraded and 10 being undegraded RNA) was selected for sequencing. Using a Ribo-Zero kit (Illumina), rRNA was subtracted from total RNA. Subsequently, mRNA was reverse transcribed to cDNA as described in Mason et al. (23). RNA (as cDNA) libraries were linker barcoded and pooled for high-throughput Illumina HiSeq 2000 paired-end sequencing at Argonne National Laboratory as described in a study by Mason et al. (22).

### Genome analyses and read mapping

The IMG/Prochlorococcus Portal (https://img.jgi.doe.gov/cgi-bin/proportal/main.cgi), hereafter referred to as ProPortal, contains 913 *Prochlorococcus* and *Synechococcus* genomes (Table S1) that were analyzed here along with the OceanDNA MAG collection of 52,000 unannotated genomes with 8,466 prokaryotic species-level clusters (18) (Table S2). ANI for all genomes was determined using Sourmash with --containment --ani --ksize 31 (52). Functional annotations targeting alkane production and consumption were identified *de novo* using Pfam (53) and KEGG (54) annotations through DRAM (55). Metatranscriptome reads were mapped to the 913 *Prochlorococcus* and *Synechococcus* genomes from ProPortal, using Bowtie2 (56). The metatranscriptome reads were also mapped to the 8,466 medium- to high-quality species-level genomes from the OceanDNA MAG catalog (18). Last, these metatranscriptomic reads were mapped to the genes annotated as *alkB*/*cyp153* in the 16 MAGs presented in Love et al. (6).

Alkane producers were defined as expressing fatty acyl-ACP reductase (*faar*, E.C. 1.2.1.80) and/or aldehyde-deformylating oxygenase (*ado*, E.C. 4.1.99.5). Primary alkane consumers were defined as expressing alkane 1-monooxygenase (*alkB*, E.C. 1.14.15.3) and/or cytochrome P450 alkane hydroxylase (*cyp153*, E.C. 1.14.15.3) in at least one sample. Although not a criterion for inclusion, secondary alkane-consumption genes, including flavin-binding monooxygenase (*fmo*, E.C. 1.14.13.8), generic cytochrome P450 (not alkane hydroxylase; *cyp450*, E.C. 1.14.14.1), rubredoxin (E.C. 1.18.1.1), and ferredoxin (E.C. 1.18.1.2), were included in the analysis to give a more comprehensive view of hydrocarbon degradation capabilities of these microbes. Reads for genome and transcript abundance were normalized by calculating RPKM values by following Thrash et al. (57). This metric normalizes read counts by both gene length and total mapped reads, which emphasizes relative differences across depths and taxonomic groups; however, it does not normalize for organism abundance, and results are interpreted qualitatively as evidence of pathway expression in natural communities rather than per-cell transcriptional rates. Low and minimal gene expression are defined here as RPKM < 5 and RPKM < 1, respectively.

## Data Availability

Metatranscriptome reads are publicly available in NCBI (PRJNA1346479). Publicly available genomes and MAGs were downloaded from https://img.jgi.doe.gov/cgi-bin/proportal/main.cgi (IMG/*Prochlorococcus* Portal) and https://doi.org/10.6084/m9.figshare.15186039.v1 (OceanDNA).

## SUPPLEMENTAL MATERIAL

Fig. S1 aem.01045-26-s0001.pdf Average nucleotide identity (ANI; genome similarity) of hydrocarbon producers.

Fig. S2 aem.01045-26-s0002.pdf Average nucleotide identity (ANI; genome similarity) of hydrocarbon consumers.

Fig. S3 aem.01045-26-s0003.pdf Heatmap of secondary alkane gene expression.

Supplemental tables aem.01045-26-s0004.xlsx Tables S1 to S5.

ASM does not own the copyrights to Supplemental Material that may be linked to, or accessed through, an article. The authors have granted ASM a non-exclusive, world-wide license to publish the Supplemental Material files. Please contact the corresponding author directly for reuse.
