# Supplementary material for: Ecosystem-scale genomic and metatranscriptomic evidence for a coupled, active short-term hydrocarbon cycle in the marine photic ocean
Source: Appl Environ Microbiol. 2026 Jul 21;92(8):e01045-26. doi: 10.1128/aem.01045-26 (PMC13488336; doi:10.1128/aem.01045-26)
Supplement: Supplementary file 2 [file aem.01045-26-s0002.pdf]

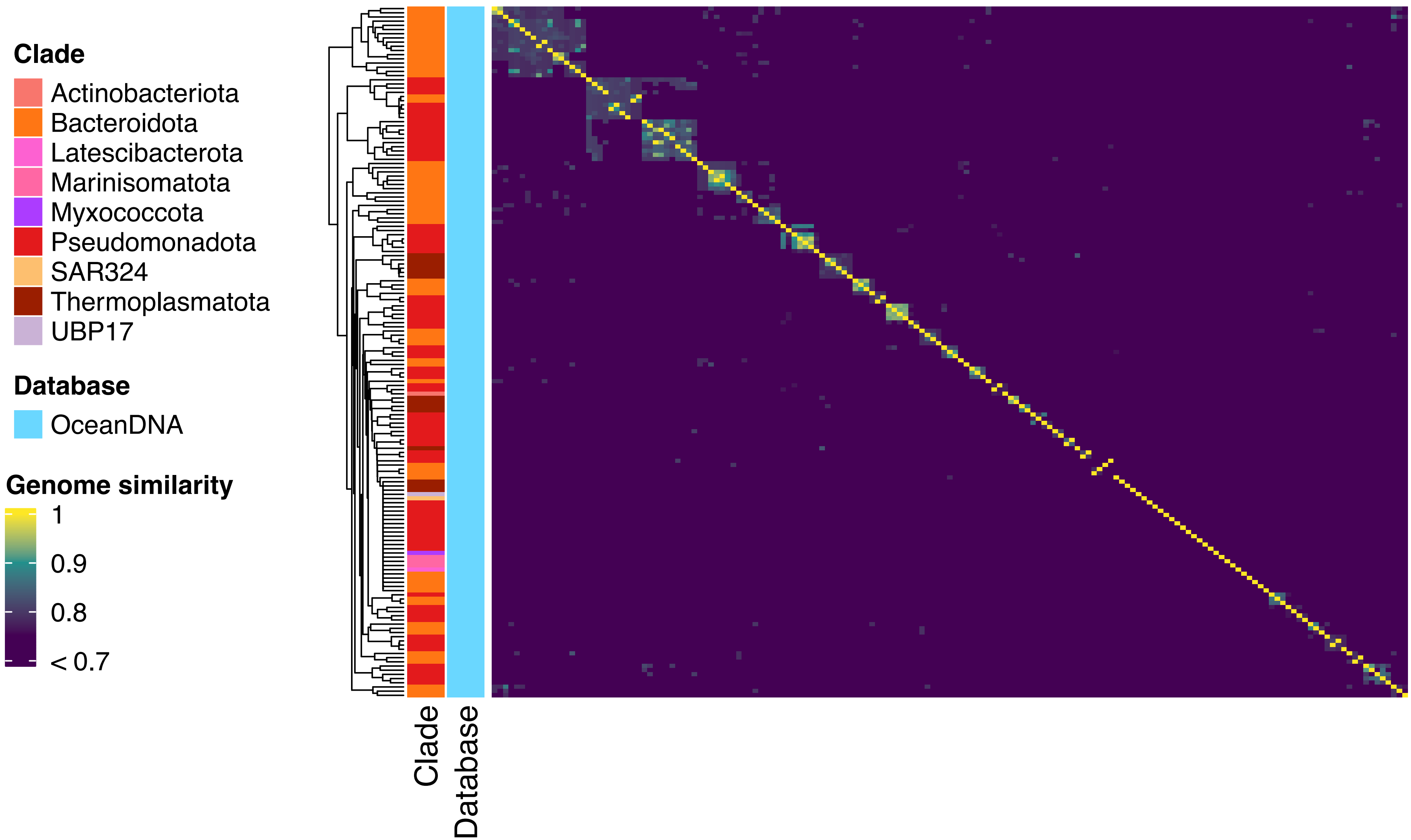

Supplemental Figure 2. Average nucleotide identity (ANI; genome similarity) of hydrocarbon consumers. The left color bar corresponds to clade and the right color bar indicates database.
