# Supplementary material for: Ecosystem-scale genomic and metatranscriptomic evidence for a coupled, active short-term hydrocarbon cycle in the marine photic ocean
Source: Appl Environ Microbiol. 2026 Jul 21;92(8):e01045-26. doi: 10.1128/aem.01045-26 (PMC13488336; doi:10.1128/aem.01045-26)
Supplement: Supplementary file 3 [file aem.01045-26-s0003.pdf]

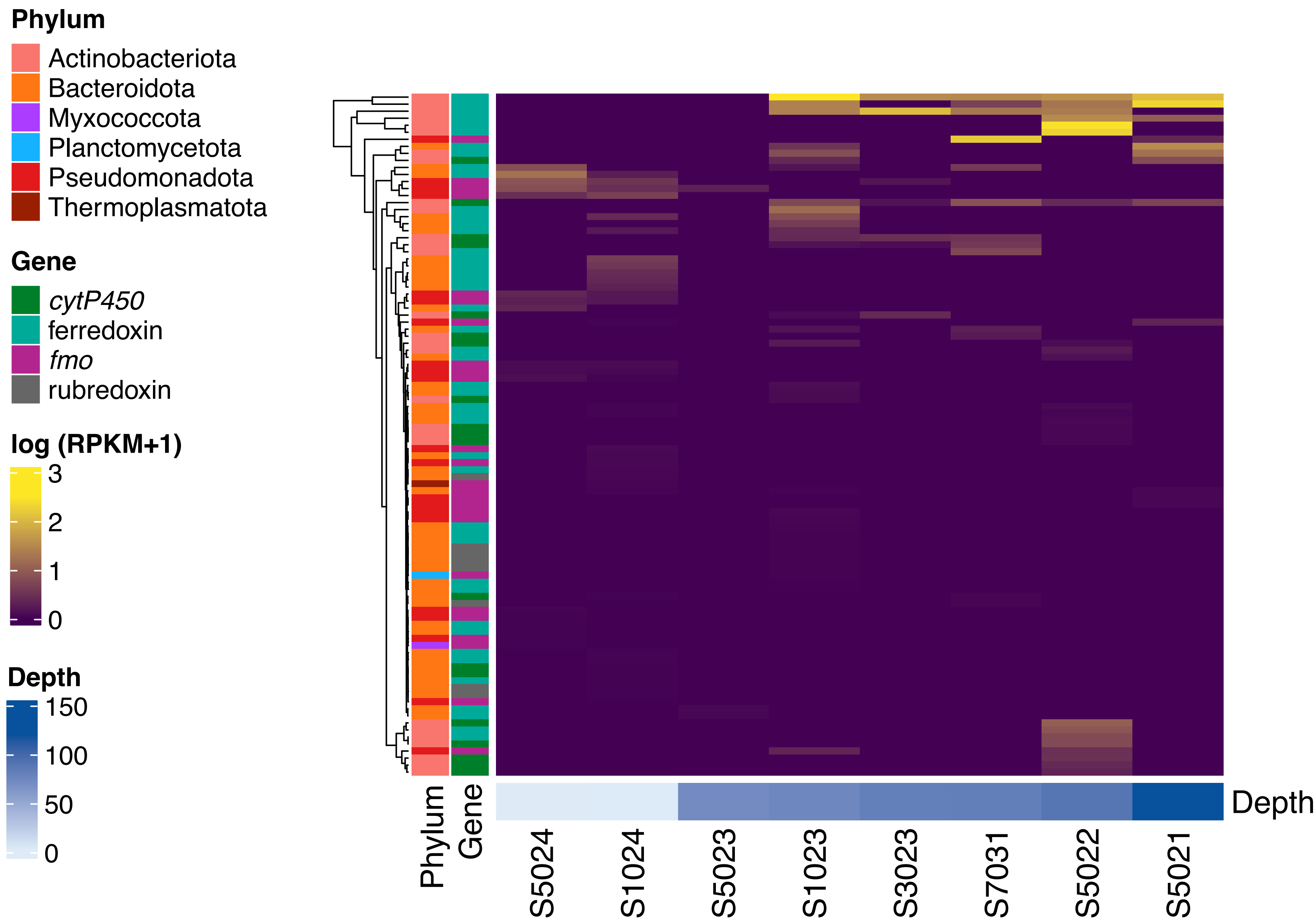

Supplemental Figure 3. Heatmap of secondary alkane gene expression. The left color bar corresponds to phylum and the right color bar indicates gene. Samples are arranged by increasing depth from left to right shown by the depth color bar.
